# The Role of Histone Protein Acetylation in Regulating Endothelial Function

**DOI:** 10.3389/fcell.2021.672447

**Published:** 2021-04-29

**Authors:** Zhi Fang, Xiang Wang, Xiaoran Sun, Wenquan Hu, Qing R. Miao

**Affiliations:** ^1^Department of Foundations of Medicine, New York University Long Island School of Medicine, Mineola, NY, United States; ^2^Department of Neurology, Tongji Medical College, Union Hospital, Huazhong University of Science and Technology, Wuhan, China

**Keywords:** epigenetic regulation, histone acetylation, acetyltransferase, deacetylase, endothelial dysfunction

## Abstract

Endothelial cell (EC), consisting of the innermost cellular layer of all types of vessels, is not only a barrier composer but also performing multiple functions in physiological processes. It actively controls the vascular tone and the extravasation of water, solutes, and macromolecules; modulates circulating immune cells as well as platelet and leukocyte recruitment/adhesion and activation. In addition, EC also tightly keeps coagulation/fibrinolysis balance and plays a major role in angiogenesis. Therefore, endothelial dysfunction contributes to the pathogenesis of many diseases. Growing pieces of evidence suggest that histone protein acetylation, an epigenetic mark, is altered in ECs under different conditions, and the acetylation status change at different lysine sites on histone protein plays a key role in endothelial dysfunction and involved in hyperglycemia, hypertension, inflammatory disease, cancer and so on. In this review, we highlight the importance of histone acetylation in regulating endothelial functions and discuss the roles of histone acetylation across the transcriptional unit of protein-coding genes in ECs under different disease-related pathophysiological processes. Since histone acetylation changes are conserved and reversible, the knowledge of histone acetylation in endothelial function regulation could provide insights to develop epigenetic interventions in preventing or treating endothelial dysfunction-related diseases.

## Introduction

Endothelial cells (ECs), mostly in the inner layer of blood vessels are co-existing with ever-changing environmental conditions, such as fluid shear stress, fluctuations of blood pressure, glucose and cytokines, nutrition availability, as well as pollutant molecules (Potente and Makinen, 2017; Sturtzel, 2017). Sensitive response to rapid changes in both internal and external cues is essential for ECs to maintain cellular homeostasis. Histone post-translational modifications (PTMs) represent crucial epigenetic mechanisms governing the ability of ECs in response to intracellular and extracellular stimuli.

To date, more than 100 different histone modifications have been detected by mass spectrometry or specific antibodies (Choudhary et al., 2014). In all eukaryotes ranging from yeast to humans, DNA is compacted more than 10,000-fold into the nucleus as chromatin. The basic structural subunit of chromatin is the nucleosome, which is comprised of 147 bp of DNA wrapped around a histone octamer core. Each nucleosome core is consisted of two copies of each of the histones: H2A, H2B, H3, and H4 (Tessarz and Kouzarides, 2014). Initially, histones were regarded as merely structural components which act as a repressive barrier to the nuclear processes requiring access to DNA. However, research over the past two decades has illustrated that histone proteins are critical for the organization and function of chromatin. Dynamic histone PTMs, largely occur at the amino-terminal tail of histones, can fundamentally regulate chromatin structure and gene transcription by spatially and temporally coordinating the shifting between condensed/transcriptionally silenced states and accessible/transcriptionally active states (Shahbazian and Grunstein, 2007). Among the diverse histone PTMs, histone acetylation has been recognized as a fundamental process that strongly affects gene expression regulation (Allfrey et al., 1964).

Growing pieces of evidence suggest that histone protein acetylation is altered in ECs under different conditions. The changes of acetylation status at different lysine sites on histone protein strongly affect EC gene expression and thus lead to endothelial dysfunction, which is involved in the pathogenesis of many cardiovascular disease processes. In this review, we will generally introduce histone acetylation and the related writers and erasers and highlight the importance of histone acetylation in regulating endothelial functions and discuss the roles of histone acetylation across the transcriptional unit of protein-coding genes in ECs under different disease-related pathophysiological processes, including vascular tone, inflammation, oxidative stress, angiogenesis, barrier function, thrombosis, and coagulation.

## Histone Acetylation

**FIGURE 1:**
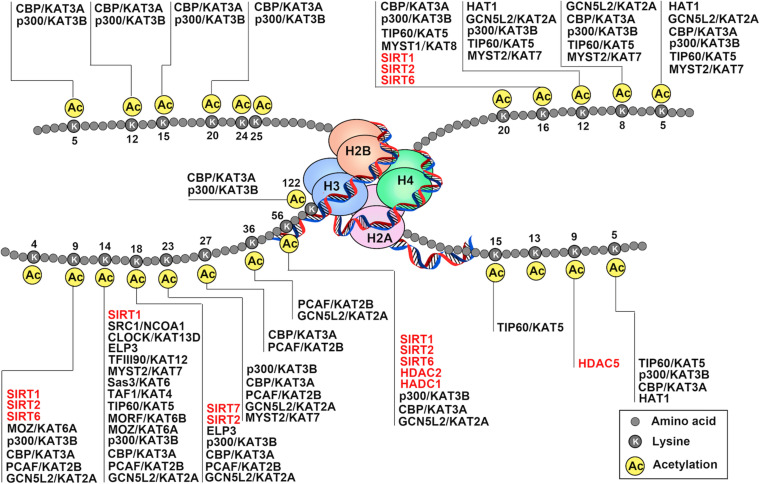
Schematic drawing of histone acetylation. DNA is wound around nucleosomes, which are composed of eight histone molecules with two copies of histones H2A, H2B, H3, and H4. Each histone molecule has a long tail rich in lysine residues (K), which are acetylation modification sites. Gene activation and repression are regulated by acetylation of core histone, and histone acetylation is mediated by coactivators (histone acetyltransferase: HATs) and corepressors (histone deacetylases: HDACs). The addition of acetyl-groups to the tails of histone core proteins leads to a relaxing state of chromatin and includes active transcription. In turn, removel of acetyl-groups from the tails of histone core proteins leads to the adoption of a condensed state of chromatin and transcriptional repression.

A common form of histone PTMs and, indeed, one of the first discovered is acetylation (Figure 1). Histone acetylation has been linked to active transcription (Phillips, 1963). Mechanically, negatively charged acetyl groups covalently added to specific lysine residues in histone can diminish the electrostatic affinity between histone proteins and DNA, thus disrupts the interaction of these histones with the DNA, leading to chromatin relaxation that enables the activation of gene transcription. Moreover, this modification can serve as docking sites for proteins involved in gene activation, such as transcriptional activators and chromatin remodeling complexes (Marmorstein and Zhou, 2014).

**FIGURE 2:**
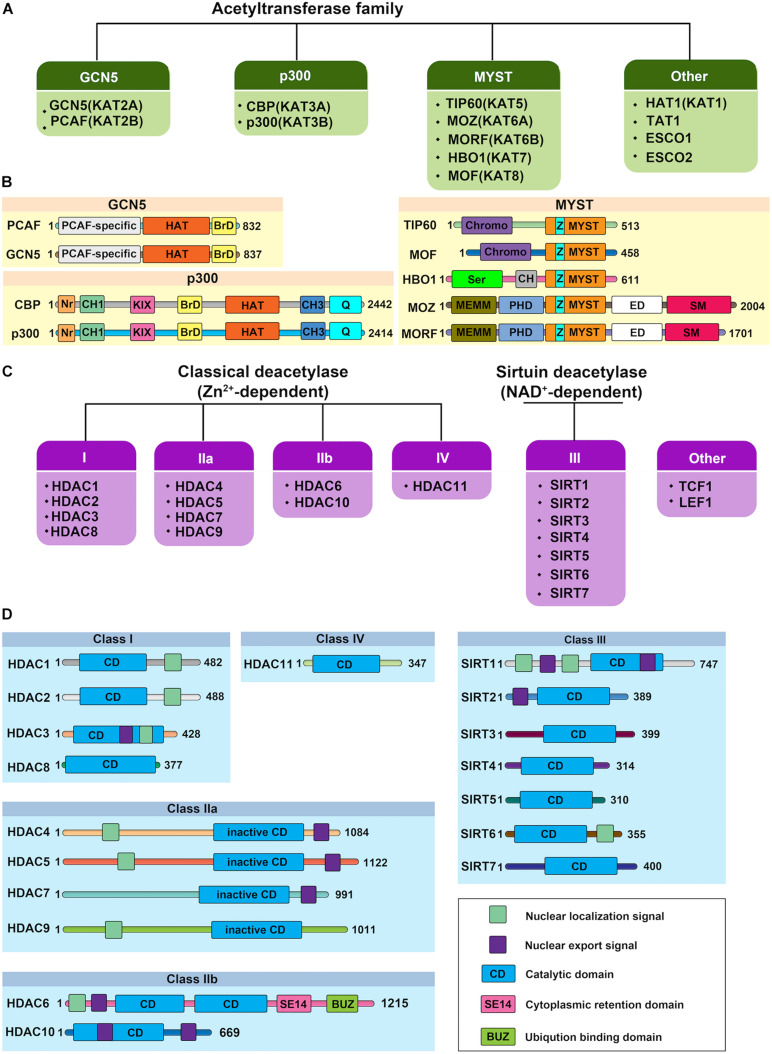
Protein structure of human histone acetylases and deacetylases. **(A)** The canonical histone acetyltransferases (HATs) are classified into three prominent families: GCN5, p300, and MYST. The other HATs are relatives dissimilar to each other. **(B)** Protein structure of human histone acetyltransferases. BrD, bromodomain; Nr, nuclear receptor-interacting box; CH, cysteine/histidine-rich module; KIX, phospho-CREB interacting module; Q, glutamine-rich domain; HAT, the lysine acetyltransferase catalytic domain; Chromo, chromodomain; Ser, serine-rich domain; NEMM, N-terminal part of MOZ or MORF; PHD, PHD zinc finger; ED, glutamate/aspartate-rich region; SM, serine/methionine-rich domain; MYST, MYST histone acetyltransferase domain; Z: C2HC zinc finger domain within the MYST domain. Numbers indicate the length of each protein. **(C)** Histone deacetylases (HDACs) are divided into two categories: the classical Zn^2+^-dependent HDACs and NAD^+^-dependent sirtuin deacetylases. Based on their phylogenetic conservation and sequence similarities, the HDACs are further classified into five major families: Class I, Class IIa, Class IIb, Class III, and Class IV. **(D)** Protein structure of human histone deacetylases. Protein domains of HDAC isoenzymes are presented above. Most HDACs possess a nuclear localization and/or export signal. Numbers indicate the length of each protein.

Histone acetylation is a highly reversible process operated in a highly regulated manner. A lysine residue becomes acetylated by the action of the histone acetyltransferases (HATs, also referred to as “writers”), which transfer acetyl group from acetyl-coenzyme A (acetyl-CoA) onto the ε-amino group of target lysine residues. To date, 37 mammalian proteins have been suggested to possess endogenous HAT activity, which can be further classified into at least five different subfamilies based on their structural homologies: the CBP/p300, GCN5/PCAF, MYST, the nuclear receptor coactivator family, and basal transcription families (Figures 2A,B; Javaid and Choi, 2017; Sheikh and Akhtar, 2019). However, due to the lack of validated evidence of HAT activities, the latter two subfamilies will not be further discussed in this review. The reverse reaction is catalyzed by histone deacetylases (HDACs, also referred to as “erasers”). In mammals, there are 18 HDAC enzymes that can be grouped into two distinct categories with different catalytic mechanisms: Zn^2+^-dependent HDACs and NAD^+^-dependent sirtuin (Sir2 orthologs) deacetylases (Figures 2C,D). On the basis of their domain structures and sequence similarities, they can be further divided into five groups: Class I comprises HDAC1, HDAC2, HDAC3, and HDAC8; the Class IIa includes HDAC4, HDAC5, HDAC7, and HDAC9; class IIb includes HDAC6 and HDAC10; Class III comprise SIRT1, SIRT2, SIRT3, SIRT4, SIRT5, SIRT6, and SIRT7; and the Class IV protein HDAC11 (Seto and Yoshida, 2014; Zhao et al., 2018). Just as histone acetylation is accomplished by “writers” and “erasers,” their actions to govern DNA transcription are mediated by “readers.” According to domain structure, various “readers” can be roughly classified into three categories, including BRD, double PHD finger (DPF), and YEATS domains (Gillette and Hill, 2015; Gong et al., 2016). Emerging evidence has also highlighted the importance of acetylation readers, and some have been implicated in physiological and pathological processes of ECs.

### Histone Acetyltransferases- Writers

#### GCN5/PCAF Family

General control non-derepressible 5 (GCN5, also called KAT2A) was one of the first proteins found to possess HAT activity (Mutlu and Puigserver, 2020). It was also the first histone acetyltransferase confirmed to link histone acetylation to transcriptional activation (Verdin and Ott, 2015). Following its discovery, some other HATs, including PCAF, which contain similar domain structures, have also been identified and consist of the GCN5/PCAF subfamily of HAT proteins. This family of HAT proteins contains PCAF-specific N-terminal domains, a HAT domain of around 160 residues, and a conserved bromodomain within the C-terminal, responsible for recognizing and binding acetyl-lysine recruiting transcriptional machinery to regulate gene expression (Downey, 2020).

**FIGURE 3:**
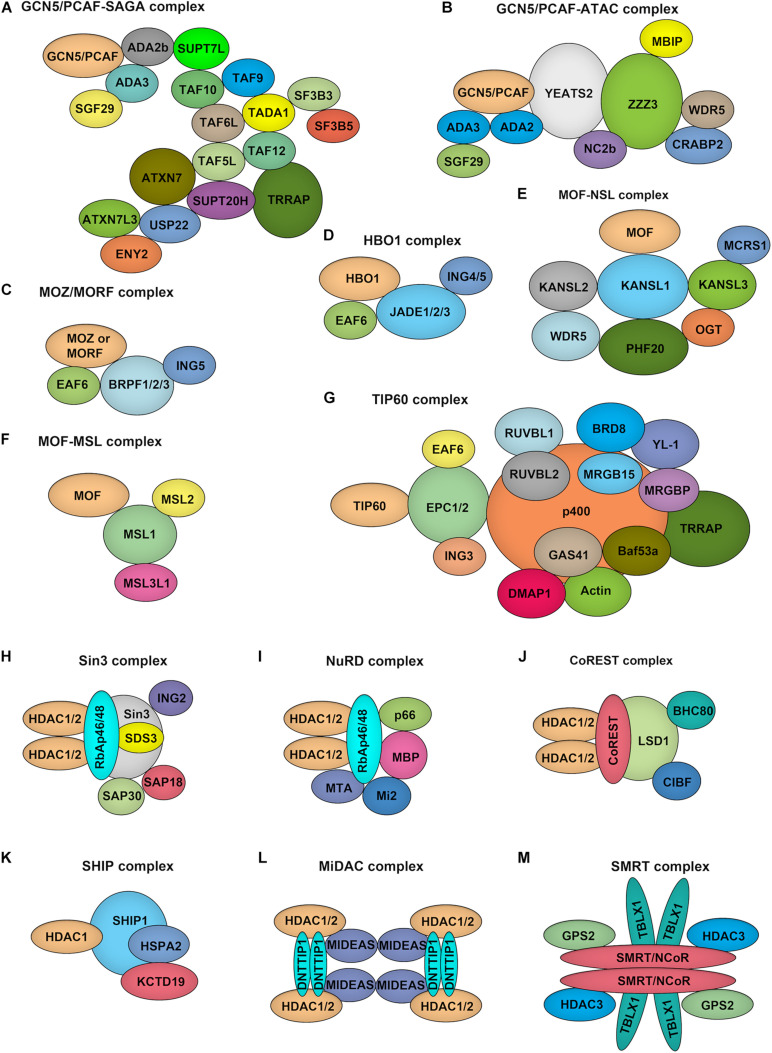
Composition of HATs and HDACs containing multiprotein complexes. **(A–G)**. Depiction of the major histone acetyltransferase complexes: GCN5/PCAF-SAGA **(A)** and GCN5/PCAF-ATAC **(B)**, MOZ/MORF-ING5 **(C)**, HBO1-JADE **(D)**, MOF-NSL **(E)**, MOF-MSL **(F)**, TIP60 **(G)**, which contains two HATs. **(H–M)**, Overview of class I HDAC containing complexes. Sin3 complex **(H)**: The Sin3 complex is comprised of HDAC1 and HDAC2, delivering enzymatic activity and the structural components Sin3, RbAp46, RbAp48, SAP18, SAP30, and SDS3. NuRD complex **(I)**: RbAp46, RbAp48, HDAC1, and HDAC2 are also found in the NuRD complex, together with the NuRD specific factors MTA, Mi-2, p66, and MBD. CoREST complex **(J)**: The CoREST complex consists of CoREST, CIBF, BHC80, HDAC1, HDAC2 and is targeted to DNA via the REST protein. SHIP complex **(K)**: The SHIP complex was identified recently as a testis specific complex containing SHIP1, HDAC1, HSPA2, and KCTD19. MiDAC complex **(L)**: The mitotic deacetylase complex (MiDAC) is a recently identified histone deacetylase (HDAC) complex using proteomics in cells that are blocked in mitosis by nocodazole. MiDAC contains HDAC1/2, a protein called DNTTIP1 (deoxynucleotidyltransferase terminal-interacting protein 1), and the MIDEAS (mitotic deacetylase-associated SANT domain protein) co-repressor protein. SMRT/NCoR complex **(M)**: The core of the complex consists of SMRT/NCoR bound to TBL1 (transducin beta-like protein 1), GPS2 (G protein pathway suppressor 2), and HDAC3 proteins.

GCN5 and PCAF both function as part of unique multi-subunit complexes, where they act as the catalytic HAT domain. For example, GCN5/PCAF is found in the complexes of Spt-Ada-GCN5 acetyltransferase (SAGA) and Ada Two A Containing (ATAC) (Figures 3A,B; Helmlinger and Tora, 2017). While the HAT module within the SAGA complex preferentially acetylates histone H3K9 and H3K14 lysine residues *in vivo*, GCN5 within the ATAC complex mainly acetylates H4K5, H4K12, and H4K16 (Spedale et al., 2012). Although the exact function of the ATAC complex remains elusive, it has been well demonstrated that following transcriptional activation, GCN5–SAGA mediates H3 acetylation throughout the gene body recruitment of the ATP-dependent chromatin remodeler SWI/SNF to target genes, which initiates a cascade of chromatin remodeling events that promotes gene transcription (Helmlinger et al., 2020).

#### CBP/p300 Family

CREB-binding protein [CREB (cyclic-AMP response element-binding protein) binding protein, CBP] and its close paralog EP300 (E1A binding protein p300) consist of another major group of HATs, which are now collectively referred to as CBP/p300 because of extensive structural homology and functional redundancy (Breen and Mapp, 2018). Both proteins are pivotal transcriptional coactivators integrating and coordinating transcriptional activation (Lipinski et al., 2019).

The members of this family are large proteins (with about 2,400 amino acids) that do not have defined molecular complexes but harbor multiple functional domains, including a considerably larger HAT domain of about 500 residues, a bromodomain to capture acetyl-lysine, and some other functional domains including the nuclear receptor-interacting box, cysteine/histidine-rich module, phospho-CREB interacting module, and glutamine-rich domain that can regulate enzyme activity and mediate diverse protein-protein interactions (Figure 2B; Bordoli et al., 2001; He et al., 2021). CBP/p300 facilitates the gene transcription not only through acetylation of histones and transcription factors but also acts as “hubs” that link chromatin-modifying proteins and basal transcriptional machinery (Rando and Chang, 2009). More importantly, recent advances reveal that CBP/p300 can be recruited by enhancers and mediate the acetylation of H3K18 and H3K27, which stimulates the establishment of an active enhancer state and facilitates transcriptional activation (Heintzman et al., 2007; Hnisz et al., 2013; Arner et al., 2015). For example, p300 was found to locate to the tissue-specific enhancers and drive the expression of adjacent genes in the forebrain or limb tissues during mouse embryonic development at a specific time point (Visel et al., 2009). Given that dynamic regulation of enhancers at specific sets of genes involving cellular differentiation, CBP/p300 can orchestrate the transcription of a series of developmentally essential genes in a temporal-specific and cell type-specific manner.

#### MYST Family

The MYST family of HAT proteins was first reported in 1996 (Reifsnyder et al., 1996), originally named for its founding members in yeast and mammals. The family currently comprises five human HATs: MOZ (MYST3/KAT6a), MORF (MYST4/KAT6b), HBO1 (MYST2/KAT7), MOF (MYST1/KAT8), and TIP60 (HTATIP/KAT5). They are defined together by the presence of a highly conserved 370 residue MYST domain, which consists of an acetyl-CoA-binding motif homologous to the canonical acetyl-CoA binding domain of GCN5/PCAF family and a C2HC zinc finger domain (Thomas and Voss, 2007). Besides the MYST domain, many members contain additional structural features, such as cysteine-rich, zinc-binding domain within the HAT regions and N-terminal chromodomains (Figure 2B; Wiesel-Motiuk and Assaraf, 2020). Based on additional notable domain regions, this family of HATs can be further classified into three subgroups: (1) MOZ and MORF, (2) MOF and TIP60, as well as (3) HBO1 alone. For example, MOZ and MORF contain two PHD-type zinc finger domains, while MOF and TIP60 are characterized by a conserved chromodomain (Santos-Rosa and Caldas, 2005).

The MYST family proteins usually form complexes with other transcriptional cofactors to coordinate transcriptional activation and transcriptional silencing. MOZ and MORF were co-purified from HeLa cells in the ING5 complex, which is consisted of MOZ/MORF, EAF, ING5, and BRPF1/2/3 (Figure 3C; Pillus, 2008). This complex can localize to the promoter regions of target genes to acetylate H3K9 and H3K23, leading to transcriptional activation. MOF occurs in two complexes identified as the MSL (male-specific lethal) complex and the NSL (non-specific lethal) complex (Figures 3E,F; Feller et al., 2012). Either MOF–MSL complex or MOF-NSL complex can trigger H4K16 acetylation at the promoter region to promote transcription, and, most intriguingly, the MOF-NSL complex can be recruited to H3K4 dimethylation positive promoters where it acetylates H4K16. In contrast, the MOF-MSL complex strongly binds to H4K20 monomethylation positive promoters in active gene bodies. TIP60 exists in a multi-subunit protein complex called NuA4 (Figure 3G; Xu et al., 2016), which shows an affinity for multiple histone acetylation sites, including H3K14, H3K27 H3K18, and H3K27. HBO1 functions in the context of a multi-protein complex called HBO1-JADE (Figure 3D), which contains ING4/5, MEAF6, and JADE1/2/3 for H3K14 acetylation throughout the gene body as well as promoters (Saksouk et al., 2009). This complex also shows an affinity for H3K4 dimethylation and trimethylation positive regions.

### Histone Deacetylases—Erasers

#### Zn^2+^-Dependent HDACs

Among the zinc-dependent HDACs, Class I, II, and IV HDACs share a conserved HDAC domain (Figure 2D). However, they have quite different characteristics. Class I HDACs majorly localize to the nucleus of mammalian cells with robust deacetylase activity. These family proteins usually function as the catalytic core within multi-subunit transcriptional repressor complexes. HDAC1/2 exist in the complexes of NuRD (nucleosome remodeling and deacetylase), SHIP (a testis-specific protein possessing putative DNA binding and chromatin remodeling properties), SIN3 (switch-independent 3), CoREST (corepressor of REST), and MiDAC (mitotic deacetylase) while HDAC3 primarily acts in NCOR1/SMRT repressor protein complex (Figures 3H–M; Kelly and Cowley, 2013; Millard et al., 2017). These complexes play essential roles for Class I HDACs to recruit them to specific target gene regions and activate their enzymatic activity. Due to the absence of a catalytic residue in the HDAC domain, class IIa HDACs, including HDAC4, HDAC5, HDAC7, and HDAC9, possess weak deacetylase activity. However, they still can serve as transcriptional repressors by forming complexes with NCOR1/SMRT (Kelly and Cowley, 2013). Also, the function of class IIa HDACs toward histone proteins is under the control of the shuttling between the cytoplasm and nucleus. Distinguished from other zinc-dependent HDACs, the class IIb family member HDAC6 is a unique member of the HDAC family that not only deacetylates histone but also targets cytoskeleton structure, including α-tubulin and cortactin (Li T. et al., 2018). For the other class IIb HDACs, HDAC10, although it can form a repressor complex with SMRT, has relatively low lysine deacetylase activity but is a robust polyamine deacetylase (Fischer et al., 2002; Hai et al., 2017). As the exclusive class IV HDAC, HDAC11 is the newest member of the HDAC family and shares the catalytic domain both of class I and II HDACs. However, it has been reported that the defatty-acylase activity of HDAC11 is over 10,000 times more than its deacetylase activity (Liu et al., 2020).

#### NAD^+^-Dependent Sirtuin Deacetylases

Sirtuin deacetylases, which have a conserved nicotinamide adenine dinucleotide (NAD)-binding and catalytic domain, are NAD^+^-dependent lysine deacetylases or ADP-ribosyltransferases. They were first identified in the yeast, referred to as the class III histone deacetylases (Carafa et al., 2016; Kida and Goligorsky, 2016). To date, seven different SIRT proteins have been identified in mammals. However, growing evidence suggests several members, including SIRT4, SIRT5 have weak deacetylase activity. Instead, extensive studies have clearly shown that SIRT proteins can catalyze the removal of diverse acyl groups from target proteins such as malonyl, succinyl, propionyl, and long-chain fatty acyl (Bosch-Presegue and Vaquero, 2015).

Besides the common deacetylase domain, SIRTs contain quite different N- and C-terminal structural features, which may explain their specified subcellular localization and functions: SIRT1, SIRT2, and SIRT6 can shuttle from nucleus to the cytoplasm; SIRT3, SIRT4, and SIRT5 are majorly mitochondrial proteins; SIRT7 localizes to nuclear and nucleolar compartment (Figure 2B; D’Onofrio et al., 2018; Singh et al., 2018).

## Role of Endothelial Histone Acetylation in Multiple Physiological Processes

### Vascular Tone

Nitric oxide (NO) is a vessel dilator regulating vascular tone (Balligand and Cannon, 1997; Alderton et al., 2001). The production of NO is regulated by the activation of nitric oxide synthase (NOS). Endothelial NOS (eNOS, also called NOS3) is constitutively expressed in vascular ECs (Wilcox et al., 1997) and plays a key role in vascular wall homeostasis and regulation of vascular tone (Balligand and Cannon, 1997; Wilcox et al., 1997; Alderton et al., 2001). Fish et al. (2005) reported that the expression of eNOS is controlled by an EC-specific histone code. Different from non-ECs, the endothelial nucleosomes that encompassed the eNOS core promoter and proximal downstream coding regions are highly enriched in acetylated histones H3K9, H4K12, and di- and tri-methylated H3K4, which are selectively associated with functionally competent RNA polymerase II complexes in ECs (Fish et al., 2005).

Proper expression of eNOS, which is required for maintaining the normal endothelial function, is regulated by histone acetylation under multiple stimuli (Table 1). Laminar shear stress (SS) has an acute stimulation of eNOS mRNA transcription (Dimmeler et al., 1999; Fleming and Busse, 1999; Boo et al., 2002). Chen et al. (2008) reported that shear stress increases p300 histone acetyltransferase activity by 2.5-fold and stimulates acetylation of histones H3 and H4 at the promoter region of the eNOS shear stress response element (SSRE) and extended 3′ toward the eNOS coding region, resulting in the opening of chromatin at the SSRE and rapid transcriptional increase of eNOS. Different from shear stress, Fish et al. (2010) demonstrated that hypoxia causes a rapid decrease in the transcription of the eNOS gene. Hypoxia rapidly evicts histone 2A (H2A.Z) from eNOS proximal promoter, and after longer durations of hypoxia exposure, acetylation of histone H3K9, H3K14, H4K5, H4K8, H4K12, and methylation of histone H3K4 decrease on eNOS proximal promoter (Fish et al., 2010). However, there is a controversy on hypoxia-induced histone acetylation mediating eNOS expression. In a rat model of hypoxia-induced persistent pulmonary hypertension of the newborn (PPHN), eNOS expression in pulmonary vascular ECs exhibits an increase with significantly higher levels of acetylated histone H3 and H4 at the proximal promoter of the eNOS gene (Xu et al., 2010). Postberg et al. (2015) also reported the increased acetylation of histone H3K9 and H2A.Z and eNOS expression after hypoxia. Chao et al. (2018) further reported that early hypoxia exposure (neonatal exposure to hyperoxia) leads to histone modification patterns as H2A.Z and H3K9 hyperacetylation and a substantial increase of the baseline expression of eNOS and STAT3. This histone modification pattern persists and becomes a signature that alters the response of ECs exposed to hypoxia later (Chao et al., 2018). In contrast, eNOS expression is decreased in another PPHN model induced by prenatal ductus arteriosus constriction in lamb. The decreased expression of eNOS is associated with DNA CpG methylation in the gene promoter region accompanied by decreased Sp1 occupancy, H4K12 acetylation, and increased H3K9 trimethylation around Sp1 binding sites (Ke et al., 2018). In human umbilical artery ECs (HUAECs), inhibition of HDAC activity using HDAC inhibitor Trichostatin A (TSA) leads to rapidly increased histone acetylation at the eNOS promoter and increases eNOS mRNA levels (Yang et al., 2012; Postberg et al., 2015). Histone H3K9 acetylation at the eNOS gene transcription start site is directly correlated with eNOS mRNA levels (Postberg et al., 2015). However, HDAC inhibition with TSA decreases eNOS mRNA while paradoxically increasing its promoter activity, suggesting a more complicated mechanism involving HDAC and histone acetylation mediated eNOS mRNA expression (Rossig et al., 2002). The suppression effect of HDAC inhibitor TSA on eNOS expression was further confirmed by Bogaard et al. (2011). Similarly, HDAC inhibitor vorinostat was reported to reduce eNOS expression more than 50% in a mouse model of diabetic nephropathy (Advani et al., 2011), and class I-specific HDAC inhibitor Valproic Acid (VPA) was demonstrated to decrease eNOS expression in HUVECs as well (Murugavel et al., 2018).

**TABLE 1:** Histone acetylation involved in the expression of vascular tone mediators.

Stimuli	Histone acetylation	HAT or HDAC involved	Targeting	References
Laminar shear stress	H3ac, H4ac	p300	eNOS (SSRE region)	Chen et al., 2008
Hypoxia	H2A.Zac, H3K9, H3K14, H4K5, H4K8, H4K12		eNOS	Fish et al., 2010
	H3ac, H4ac		eNOS	Xu et al., 2010
	H3K9ac, H2A.Zac		eNOS (STAT3 binding)	Postberg et al., 2015; Chao et al., 2018
Persistent pulmonary hypertension of newborn	H4K12ac		eNOS (Sp1 binding)	Ke et al., 2018
27nt small RNA	H3K9ac, H3K12ac, H3K23ac, H4K8ac, H4K12ac	HDAC3	eNOS	Zhang et al., 2008
DJ-1/park7	H3ac	HDAC1	eNOS	Won et al., 2014
Athero protective pulsatile shear stress	H3K27ac		ITPR3 (KLF4 binding)	He et al., 2019
Pathogenic factors of hypertension	H3K9ac	SIRT6	Nkx3.2	Guo et al., 2019
Pathogenic factors of heart failure	H3K56ac	SIRT3, p300	eNOS, arginase II	Su et al., 2020
Maternal nutrient restriction	H3K9ac, H3K18ac, H4ac		ET-1 (HIF-1a binding)	Xu et al., 2013
Shear stress	H3K14ac	CBP	c-fos, c-jun	Illi et al., 2003

The 27nt small RNA, generated from 27nt repeat polymorphism in eNOS intron 4, inhibits eNOS expression. In human aortic ECs (HAECs), the 27 nt small RNA duplex induces hyperacetylation in H3K8, H3K12, H3K23, and H4K9, H4K12 at the 27 nt repeat element and DNA methylation in a region approximately 750nt upstream of the intron 4 repeats of eNOS gene, which is interacted with Non-O (Non-POU Domain Containing Octamer Binding portion) and HDAC3 proteins. The latter deacetylated H3 and H4 at the eNOS core promoter, thereby reduces the expression of eNOS. Treatment with HDAC3 small interfering RNA (siRNA) significantly mitigates the 27 nt small RNA-suppressed expression of eNOS (Zhang et al., 2008). DJ-1/park7, a multifunctional protein, is reported to be implicated in the regulation of vascular contractility and blood pressure by the impairment of NO production through epigenetic inhibition of eNOS expression. HDAC1 recruitment in the eNOS promoter regions is significantly increased in ECs isolated from DJ-1/park7^–/–^ mice compared with control mice. Moreover, the acetylation of H3 histones in the eNOS promoter regions is decreased in DJ-1/park7^–/–^ mice. These DJ-1/park7-dependent epigenetic characteristics in mouse ECs are also appreciated in HUVECs (Won et al., 2014).

Besides by directly binding of acetylated histone proteins on the eNOS promoter region, eNOS expression is also regulated by indirectly histone acetylation-dependent regulation. Atheroprotective pulsatile shear stress induces KLF4-dependent binding of acetylated H3K27 at the ITPR3 promotor region and increases chromatin accessibility, RNA polymerase II recruitment, and ITPR3 transcription. The ITPR3 induction further elevates the expression of eNOS and NO bioavailability (He et al., 2019).

Histone deacetylase SIRT6 in ECs has pleiotropic protective actions, which include promoting endothelium-dependent vasodilatation and vascular NO bioavailability. It induces the expression of GATA-binding protein 5 (GATA5), a novel regulator of blood pressure, through inhibiting NK3 homeobox 2 (Nkx3.2) transcription by deacetylating histone H3K9, thereby regulating GATA5-mediated signaling pathways to prevent endothelial injury (Guo et al., 2019). Histone deacetylase SIRT3 deletion in ECs causes cardiac remodeling and diastolic dysfunction (He et al., 2017), associated closely with vascular tone. Knockout of SIRT3 results in significant increases in p300 expression and histone H3K56 acetylation. P300 inhibitor C646 significantly reduced levels of p300 and H3K56 acetylation and increased eNOS expression (Su et al., 2020). As a product of eNOS, NO also modulates essential endothelial functions and gene expression through histone deacetylation. Mechanistically, NO induces class II HDAC4, and HDAC5 nuclear shuttling via activation of the protein phosphatase 2A (PP2A) (Illi et al., 2008).

Endothelin-1 (ET1), another critical vasoconstrictor secreted by EC, plays a crucial role in vascular tone. ET1-mediated vascular tone increases with age and contributes to the pathogenesis of hypertension (Stauffer et al., 2008). The maternal nutrient restriction increases the acetylation of histone H3K9, H3K18, and H4 and hypoxia-inducible factor-1α (HIF-1α) binding levels at the gene promoter of ET1 in pulmonary vascular ECs (PVEC) of intrauterine growth retardation (IUGR) newborn rats and continues up to 6 weeks after birth. These epigenetic changes result in endothelial dysfunction, and IUGR rats are highly sensitive to hypoxia later in life, causing more significant pulmonary arterial hypertension (PAH) (Xu et al., 2013).

Except for eNOS, shear stress (SS) modulates the expression of several other genes in ECs and regulates vascular tone (Illi et al., 2003; Iring et al., 2019). In HUVECs, SS activates ribosomal S6 kinase-2 and mitogen- and stress-activated kinase-1 protein kinases and promotes the formation of CREB/CBP transcriptional complexes thus increases the histone acetyltransferase activity and acetylates histone H3K14 within the c-fos and c-jun promoters. Consequently, shear stress increases the expression of transcription factors c-fos and c-jun and initiates the transcription of c-fos and c-jun-targeted genes in ECs (Illi et al., 2003).

### Inflammation

As an important player in the inflammation process, EC participates in multiple cytokine production and secretion and responds to cytokines stimuli through the mechanism of involving histone acetylation, as summarized in Table 2.

**TABLE 2:** Inflammation signals mediated by histone acetylation in ECs.

Stimuli	Histone acetylation	HAT or HDAC involved	Targeting	References
Dengue virus infection	H3ac, H4ac		IL-8	Bosch et al., 2002
Listeria monocytogenes infection	H4K8ac, H3K14ac	CBP	IL-8	Schmeck et al., 2005
	H4ac, H3K14ac		IL-8 (NF-κB/p65 binding)	Schmeck et al., 2006
Chlamydophila pneumoniae infection	H3ac, H4ac		Cytokines (p65/RelA binding)	Schmeck et al., 2008
Bach1	H3ac, H4ac	HDAC1	IL-8	Jiang et al., 2015
Integrin engagement	H3ac		ICAM-1, VCAM-1	Rose et al., 2005
TNF-α stimulus during blood vessel maturation	H3ac		E-selectin	Hamada et al., 2014
LDL	H3K9ac, H3K14ac		p66shc	Kim et al., 2012
oxLDL	H3K14ac, H4ac	CBP, p300, HDAC1, HDAC2	IL-8, MCP-1	Dje N’Guessan et al., 2009
Hyperglycemia or RAGE	H3K9ac		Cox2 (NF-κB binding)	Perrone et al., 2009
Lysophosphatidylcholine	H3K14ac		ICAM-1 (AP-1 binding)	Li X. et al., 2018; Lu et al., 2019
Angiotensin II	H3ac		CSF1	Shao et al., 2019
Tumor angiogenic growth factors	H3ac		ICAM-1	Hellebrekers et al., 2006
Estrogen	H3ac, H4ac	CBP	MHC II	Adamski et al., 2004
IL-1β	H3K27ac		Genomic sites occupied by NOTCH1-RBPJ	Poulsen et al., 2018
TNF-α	H3ac		Netrin-1	Passacquale et al., 2015
TNF-α or IL-1β	H3K27ac, H4ac	HDAC2	RNASE1	Bedenbender et al., 2019
Polychlorinated biphenyls	H3ac	p300, HADC1, HDAC2	IL-6, CRP, ICAM-1, VCAM-1, IL-1α, IL-1β	Liu et al., 2016
Epigallocatechin-3-gallate	H3K9ac, H3K14ac	HDAC5, HDAC7, p300, CBP	Chromatin relaxation	Ciesielski et al., 2020

Histone acetylation mediates cytokine expression and secretion in various infectious diseases. After dengue virus infection, the infected monocytes, ECs, and epithelial cells increase the secretion of interleukin 8 (IL-8) and results in an increased serum level of IL-8 significantly. The induction of IL-8 is associated with the hyperacetylation of histone H3 and H4 at the IL-8 promoter in addition to the transcription activated by NF-kB (Bosch et al., 2002). Intracellular *Listeria monocytogenes* infection induces time-dependent acetylation of histone H4K8 and H3K14 at the IL-8 promoter in HUVEC, as well as recruitment of the histone acetylase CBP (Schmeck et al., 2005). Inhibition of RhoA, Rac1, and Cdc42 in HUVEC reduces *Listeria*-promoted recruitment of NF-kB/p65 and RNA polymerase II to the IL-8 promoter, as well as acetylation of histone H4 and H3K14 at the IL-8 gene promoter (Schmeck et al., 2006). Inflammatory activation of the endothelium by *Chlamydophila pneumonia* infection is involved in the development of chronic vascular lesions. *Chlamydophila pneumonia-*induced expression of inflammatory genes (IL-6, IL-8, G-CSF, GM-CSF, MIP-1β, and IFN-γ) in ECs is regulated by Rho-GTPase–related acetylation of histone H3 and H4 (Schmeck et al., 2008). Integrin engagement, contributing to antigenotoxic effects of lung endothelium, increases the acetylation of core histone H3 at the promoters of intercellular adhesion molecule-1 (ICAM-1) and vascular cell adhesion molecule-1 (VCAM-1), similar to the effect of HDAC inhibitor TSA (Rose et al., 2005). However, the effect of HDAC inhibitors on VCAM-1 in ECs is opposite under TNF-α stimulation. Inoue et al. (2006) and Hebbel et al. (2010) reported that HDAC inhibitors TSA and suberoylanilide hydroxamic acid (SAHA) greatly reduced TNF-α-stimulated VCAM-1 expression in ECs. As another case of histone acetylation-mediated cytokine expression in HUVECs, BTB, and CNC homology 1 (Bach1), a basic leucine zipper transcription factor, recruits HDAC1 to the IL-8 promoter and decreases histone H3 and H4 acetylation at the transcription factor 4 (TCF4)-binding site of IL-8 promoter (Jiang et al., 2015). Though histone hyperacetylation is involved in promoting inflammatory cytokine production and secretion, HDAC inhibitors, generally increasing histone acetylation, have a suppressive effect on inflammatory cytokine production induced by lipopolysaccharide (LPS) or TNF-α. A reduction in IL-6 and IL-8 gene expression by HDAC inhibitors, such as butyrate, propionate, and TSA, is observed in HUVECs (Li M. et al., 2018). HDAC inhibitor butyrate also suppresses gene expression and LPS-induced secretion of pro-inflammatory genes CCL2, IL-6, and IL-1β, via enhancing histone H3K9/14 and H2A.Z acetylation and the associated upregulation of oxidative stress-protective genes IRS1, Catalase, SOD2, FOXO3A, and PGC-1α expression in human microvascular ECs (HMEC-1) (Chriett et al., 2019).

Inflammatory response changes during blood vessel maturation and this change has been demonstrated to go through histone H3 acetylation mediated E-selectin expression in response to TNF-α stimulus during blood vessel maturation (Hamada et al., 2014). In many chronic diseases, histone acetylation plays a critical role in endothelial dysfunction associated with inflammation. Hypercholesterolemia induces p66shc-mediated proinflammatory endothelial dysfunction and atheromatous plaque formation by inducing ICAM-1 and reducing thrombomodulin (TM). Mechanistically, low-density lipoprotein (LDL) promotes hypomethylation of two CpG dinucleotides and acetylation of histone H3K9 and H3K14 in the p66shc promoter, thus increasing endothelial p66shc expression (Kim et al., 2012). Similar to LDL, oxidized low-density lipoprotein (oxLDL) also has a proinflammation feature. It induces lectin-like oxidized LDL receptor-1 (LOX-1) and extracellular regulated kinases (ERK1/2)-dependent acetylation of histone H3K14 and H4 binding onto the promoters of IL-8 and monocyte-chemoattractant protein-1 (MCP-1). Consequently, oxLDL increases the expression and secretion of IL-8 and MCP-1 in ECs (Dje N’Guessan et al., 2009). Clinically used statins, which block 3-hydroxy-3-methylglutaryl coenzyme A (HMG Co-A), has a beneficial effect on atherosclerosis depending at least in part on the inhibition of the release of proinflammatory cytokines (Liao and Laufs, 2005). The use of statins alters the histone modification by reducing the recruitment of CBP/p300, NF-kB, and RNA polymerase II but increasing the binding of HDAC1 and HDAC2 at the promoters of IL-8 and MCP-1 genes (Dje N’Guessan et al., 2009). Thioredoxin-interacting protein (TXNIP) plays a causative role in diabetic vascular complications by increasing proinflammatory factor cyclooxygenase (COX)-2 expression in ECs. It induces COX-2 expression by abolishing H3K9 tri-methylation and increasing H3K9 acetylation at the proximal COX-2 promoter bearing the NF-kB-binding site (Perrone et al., 2009). However, HDAC inhibitor TSA decreases LPS-induced COX-2 expression in HUVECs, which is caused by the non-acetylation effect of TSA and may be due to JNK and p38MAPK dephosphorylation induced by TSA activated MKP-1 (Hsu et al., 2011). Lysophosphatidylcholine (LPC) induces mitochondrial reactive oxygen species-dependent site-specific H3K14 acetylation, therefore increases the binding of proinflammatory transcription factor AP-1 in the promoter of ICAM-1 and induces ICAM-1 transcription in HAECs. However, IL-35, an anti-inflammatory cytokine, suppresses LPC-induced monocyte adhesion via ICAM-1 mediated regulation (Li X. et al., 2018). The further RNA-seq and ChIP-seq analysis results demonstrated that LPC induces H3K14 acetylation in the genomic DNA that encodes LPC-induced immunity pathway genes including ICAM-1 (Lu et al., 2019).

Angiotensin II (Ang II), a causative factor for hypertension, has been documented to stimulate endothelium-derived colony-stimulating factor (CSF1), which is a key molecule controlling the production, differentiation, and function of macrophage) transcription (da Cunha et al., 2005). Shao et al. (2019) demonstrated that Ang II upregulates both acetylated H3 and tri-methylated H3K4 and downregulates dimethyl H3K9 surrounding the proximal CSF1 promoter via BRG1 interaction with histone methyltransferase SET1A and histone demethylase JMJD1A. In the tumor, another chronic disease, it has been demonstrated that tumor cells could go immune escape through histone acetylation mediated endothelial dysfunction. As a mediator of leukocyte-EC adhesion, ICAM-1 expression in tumor ECs is epigenetically silenced by promoter histone modifications (histone H3 deacetylation and histone H3K4 demethylation) while restored by HDAC inhibitor TSA (Hellebrekers et al., 2006).

Except for the production of cytokines, the responses of ECs to cytokine or inflammatory stimuli also are associated with histone acetylation. Estrogen has immunomodulatory effects and reduces the class II MHC expression, which has been demonstrated to go through attenuating the acetylation of histone H3 and H4 and the association of CBP within the class II MHC promoter (Adamski et al., 2004). Histone H3K27 acetylation is required for proinflammatory cytokine IL-1β-induced rapid recruitment of NF-κB subunit RELA to genomic sites occupied by NOTCH1-RBPJ (Poulsen et al., 2018). Netrin-1, a neuroimmune guidance cue (Boyer and Gupton, 2018), has chemorepellent action of the endothelium against monocyte chemotaxis. TNF-α-induced NF-κB activation upregulates the nuclear isoform of netrin-1 while simultaneously reducing secreted netrin-1 through histone H3 hyperacetylation. Besides, aspirin, a COX inhibitor that is widely used as an antiplatelet agent but exerts anti-inflammatory properties as well (Desborough and Keeling, 2017), also regulates the TNF-α-induced secretion of netrin-1 via epigenetic modification. Mechanistically, aspirin reduces HDAC/HAT ratio and induces higher expression of acetylated histone H3 (Passacquale et al., 2015). Upon long-term inflammation, high amounts of proinflammatory cytokines affect endothelial function by downregulating RNase1, a circulating extracellular endonuclease regulating vascular homeostasis of extracellular RNA and acting as a vessel- and tissue-protective enzyme (Fischer et al., 2007; Cabrera-Fuentes et al., 2015). In HUVECs, TNF-α, or IL-1β stimulation reduces the expression of RNase1 by inducing hypoacetylation of histone H3K27 and histone H4 via accumulation of HDAC2 to the RNASE1 promoter, while class I HDAC specific inhibitor MS275 abolishes the changes (Bedenbender et al., 2019). Lipoxin A4 (LXA4), a primary stop signal of inflammation, exerts potent bioactions by activating a specific G protein-coupled receptor ALX/FPR2. Activation of p300 restores chromatin accessibility and significantly increases ALX/FPR2 mRNA and protein levels in pulmonary artery ECs (PAECs). Changes in the histone acetylation status enhance ALX/FPR2 signaling in response to LXA4 (Simiele et al., 2016).

Environmental pollutants, such as polychlorinated biphenyls (PCBs), are also reported to induce endothelial inflammation via histone acetylation. PCB 126 exposure increases the expression of vascular inflammatory mediators, including IL-6, C-reactive protein (CRP), ICAM-1, VCAM-1, and IL-1α/β in EA.hy926 human ECs (Liu et al., 2016). Anti-inflammatory polyphenol epigallocatechin-3-gallate (EGCG), the main green tea polyphenol, is demonstrated to abolish nuclear translocation of p65, decrease chromatin binding of p65, and p300, and increase chromatin binding of HDAC1/2, therefore deacetylate histone H3 and deactivate the proinflammatory genes (Liu et al., 2016). Besides, EGCG also increases the chromatin marks of H3K9 and H3K14 acetylation as well as H3K4 and H3K9 trimethylation, presenting a broad epigenetic potential in affecting the expression and activity of epigenome modulators including HDAC5 and 7, p300, CBP, Lysine Demethylase 1A (LSD1) or KMT2A (Ciesielski et al., 2020).

### Oxidative Stress

Oxidative stress is a complicated factor affecting EC functions. ECs produce both reactive oxygen species (ROS) and anti-oxidative molecules. The proper balance is required to maintain normal endothelial function. Multiple stimuli affect the balance, and excessive ROS causes endothelial dysfunction. Among these processes, histone acetylation plays an important role, as summarized in Table 3. Inactivation of the p66Shc adaptor protein confers EC resistance to oxidative stress. However, high-glucose induces overexpression of p66Shc. It has been reported that p66Shc-deficient mice are protected against age-related and hyperglycemia-induced endothelial dysfunction. SIRT1 inhibits high glucose-induced upregulation of p66Shc via decreasing the acetylation of histone H3 binding onto the p66Shc promoter (Zhou et al., 2011). The p66Shc gene overexpression induced by high-glucose is epigenetically regulated by promoter CpG hypomethylation (Paneni et al., 2012) and histone H3K9 acetylation along with enhanced transcriptional factor p53 binding (Mishra et al., 2019). Besides mediating the endothelial response to oxidative stress, p66Shc is also emerging as a key molecule responsible for ROS generation and vascular damage. Methyltransferase SUV39H1, demethylase JMJD2C, and acetyltransferase SRC-1 are found to be associated with reduced di- and tri-methylation as well as acetylation of H3K9 on the p66Shc promoter in visceral fat arteries (VFA), which results in the elevated expression of p66Shc and ROS-related endothelial dysfunction in VFA (Costantino et al., 2019).

**TABLE 3:** Histone acetylation involved in endothelial mediated oxidative stress.

Stimuli	Histone acetylation	HAT or HDAC involved	Targeting	References
Hyper glycemia	H3K9ac	SIRT1, SRC-1	p66shc (p53 binding)	Zhou et al., 2011; Paneni et al., 2012; Costantino et al., 2019; Mishra et al., 2019
H2O2	H3K14ac, H4K16ac	SIRT1	p53	Jamal et al., 2014
HDACi scriptaid	H4ac, H3K27ac	HDAC3	Nox4 (c-jun binding)	Siuda et al., 2012
HDAC inhibitors	H3K27ac		SOD3	Zelko and Folz, 2015
Exendin-4	H3ac	HDAC activity	SOD3	Yasuda et al., 2016
CAPE	H3ac	HDAC1	SOD3 (MEF2A binding)	Ohashi et al., 2017
Hyper glycemia	H3K9ac, H3K27ac	HDAC2	SOD2	Hou et al., 2018
Oxidative stress	H4K16ac	SIRT1	Antioxidant genes (FoxO3a and PGC-1 binding)	Olmos et al., 2013

Hydrogen peroxide (H_2_O_2_), ROS inducer, upregulates p53 and acetylation of H3K14 and H4K16 due to the decreased activity of SIRT1. Paeonol, a phenolic compound isolated mainly from Moutan cortex, the root bark of the Chinese Peony tree, significantly increases the deacetylase activity of SIRT1 in HUVECs. Pretreatment with paeonol substantially decreases the H_2_O_2__–_upregulated levels of acetylated H3K14 and H4K16 (Jamal et al., 2014). NADPH oxidase (NOX) promotes ROS production in ECs. In response to hypoxia-reoxygenation (HR), chromatin remodeling protein BRG1 is recruited to the NOX promoter region and induces acetylation of histones H3 and H4 and methylation of histone H3K9 surrounding the NOX promoter (Li Z. et al., 2018). In human EA.hy926 ECs, siRNA-mediated knockdown of HDAC3 leads to a significant downregulation of NOX family member Nox4 expression. Also, HDAC inhibitor Scriptaid leads to the enhanced acetylation of histone H4 and H3K27, and decreases Nox4 transcription by preventing the binding of transcription factor c-Jun and RNA polymerase IIa to the Nox4 promoter due to hyperacetylation-mediated steric inhibition (Siuda et al., 2012).

Extracellular superoxide dismutase (EC-SOD, SOD3) is the major antioxidant enzyme present in the vascular wall. The expression of antioxidant genes in PAECs is regulated by epigenetic mechanisms (Zelko et al., 2011). HDAC inhibitor Scriptaid-mediated augmentation of SOD3 expression is associated with increased histone H3K27 acetylation and H3K4 trimethylation at the promoter of the SOD3 gene (Zelko and Folz, 2015). Exendin-4, a glucagon-like peptide-1 receptor agonist, significantly induces the expression of SOD3 in human retinal microvascular ECs (HRECs) by decreasing HDAC activity and enhancing the status of histone H3 acetylation at the SOD3 proximal promoter and (Yasuda et al., 2016). Moreover, Caffeic acid phenethyl ester (CAPE) increases the transcription of SOD3 through HDAC1-regulated histone acetylation in HRECs (Ohashi et al., 2017). Except for SOD3, SOD2 (Superoxide Dismutase 2, also called MnSOD) is also regulated by histone acetylation. Diabetes-induced HDAC2 upregulation diminishes the acetylation of histone H3K9 and H3K27, therefore, reduces SOD2 expression. The reduced SOD2 expression promotes oxidative stress and contributes to diabetes-related endothelial dysfunction (Hou et al., 2018). SIRT1 activates the expression of antioxidant genes in ECs by deacetylating and increasing the transcription factors FOXO3a and PGC-1α, however reducing H4K16 acetylation and increasing elongating Pol II recruitment occurring at these genes (Olmos et al., 2013).

### Angiogenesis

Angiogenesis is a process of new blood vessel formation, which is the well-characterized function of ECs. As summarized in Table 4, histone acetylation is found to be crucial in regulating angiogenesis.

**TABLE 4:** Histone acetylation associated with angiogenesis.

Stimuli	Histone acetylation	HAT or HDAC involved	Targeting	References
Angiogenic signals	H3K56ac	CBP	VEGFR1	Dutta et al., 2010
VEGF stimulation	H3ac	PCAF	VEGFR1, VEGFR2, ANGPT2 (E2F1 binding)	Pillai et al., 2010
VEGF stimulation	H3K27ac	p300	Angiogenic genes	Zhang et al., 2013
SerRS	H4K16ac	SIRT2	VEGFA (c-myc binding)	Shi et al., 2014
VEGF stimulation	H3K9ac, H3K14 ac, H4ac	p300	VE-cadherin (SRF binding)	Shu et al., 2015
Osteoporosis (ZEB1 loss)	H3K4ac, H3K14ac, H3K18ac	p300, CBP	Dll4, NOTCH1	Fu et al., 2020
NF-kB signal	H3ac, H4ac	p300, HDAC1	cFLIP, NFAT	Aurora et al., 2010
CO	H3K9ac, H3K18ac, H3K23ac	HDAC3 (implied)	Angiogenic genes	Li et al., 2014
KAT7 KO	H3K14ac, H4ac	KAT7	VEGFR2	Yan et al., 2018
VEGF stimulation and HDAC inhibitors	H3K9ac	HDAC1 (implied)	Angiogenic genes	Jin et al., 2011
HDAC7 KO	H3ac	HDAC7	AKAP12	Turtoi et al., 2012

In response to angiogenic signals, histone cell cycle regulation defective homolog A (HIRA) is induced in ECs and mediates the incorporation of CBP acetylated H3K56 at the chromatin domain of VEGFR1, which then induces transcription of several angiogenic genes in ECs (Dutta et al., 2010). Upon VEGF stimulation, angiogenic genes VEGFR1, VEGFR2, and angiopoietin 2 (ANGPT2) in HUVECs are induced via increased promotor binding of transcription factor E2F1, which is correlated with the recruitment of p300 and PCAF and acetylation of histones and E2F1 (Pillai et al., 2010). Zhang et al. (2013) also reported that the greatest change of H3K27 acetylation (H3K27ac) is associated tightly with p300 in VEGF-stimulated HUVECs. Dynamic H3K27ac deposition and associated changes in chromatin conformation require p300 activity instead of altered nucleosome occupancy or changes in DNase I hypersensitivity (Zhang et al., 2013). Moreover, human metallothionein 1G (hMT1G) is upregulated upon VEGF stimulation in HAECs. VEGF stimulation leads to dissociation of HDAC1 from the promoter, acetylation of histones, and increased binding of transcription factor E2F1. The upregulation of hMT1G contributes to the proangiogenic functions of VEGF (Joshi et al., 2005). Seryl-tRNA synthetase (SerRS) plays an essential role in regulating vascular development by acetylated histone-mediated regulation of VEGFA expression. In particular, SerRS blocks the binding of c-Myc, the major transcription factor promoting VEGFA, to VEGFA promoter and recruits SIRT2 to erase c-Myc-promoted acetylation of H4K16 so as to intervene c-Myc-dependent VEGFA expression (Shi et al., 2014).

Vascular endothelial cadherin (VE-cadherin) is the primary determinant of EC junction integrity during vascular development and angiogenesis. During angiogenesis and neovascularization processes, VEGF induces the binding of myocardin-related transcription factor-A (MRTF-A) to the Serum Response Factor (SRF)-binding site within the VE-cadherin promoter. MRTF-A and p300 synergistically augment VE-cadherin expression by enhancing acetylation of H3K9, H3K14, and H4 at the SRF-binding site within the VE-cadherin promoter (Shu et al., 2015). Osteoporosis-associated downregulation of zinc-finger transcription factor ZEB1 in ECs reduces CBP/p300 mediated acetylation of H3K4, H3K14, and H3K18 within the promoters of Dll4 and Notch1, thereby epigenetically suppressing Notch signaling, a critical pathway that controls bone angiogenesis and osteogenesis (Fu et al., 2020).

NF-kB signal is induced by antiangiogenic molecules and has a dual effect on downstream transcription through histone acetylation regulation. On the one hand, NF-kB acts as an activator of proapoptotic Fas ligand via recruiting p300 and acetylated histones H3 and H4 at its promotor. On the other hand, NF-kB acts as a repressor of antiapoptotic cFLIP [cellular FLICE (FADD-like IL-1β-converting enzyme)-inhibitory protein] via reducing the HDAC1 and p300 coordinated histone acetylation and recruitment of transcription factor NFAT critically required for cFLIP transcription (Aurora et al., 2010). Administration of low, safe doses of exogenous carbon monoxide (CO) was demonstrated to enhance EC migration and angiogenesis in the acetylated H3K9, H3K18, and H3K23 dependent manner, nuclear receptor Rev-erbα, a haem-containing transcription factor, responds to CO stimulation, recruits the HDAC/nuclear Receptor Corepressor (N-CoR) complex, and thus suppresses the transcription of genes responsible for EC migration and angiogenesis (Li et al., 2014).

Histone acetyltransferase and deacetylase are potentially involved in regulating angiogenesis through the alteration of histone acetylation. KAT7 participates in regulating VEGFR-2 transcription by promoting the acetylation of H3K14 and H4 and RNA polymerase II binding. Depletion of KAT7 reduces VEGFR2 expression and disrupts angiogenic potential in HUVECs (Yan et al., 2018). HDAC inhibitor VPA results in hyperacetylated H3K9 and greatly enhances the VEGF-promoted spheroid sprouting of HUVECs (Jin et al., 2011). HDAC inhibitor LBH589 induces the acetylation of histone H3 and non-histone protein α-tubulin and induces G2-M cell cycle arrest, inhibits the VEGF-induced expression of ANGPT2, survivin, HIF-1α, and CXCR4 possibly due to histone acetylation modulated upregulation of tumor suppressor genes, such as p53 and VHL (Von Hippel–Lindau) (Qian et al., 2006). Novel HDAC inhibitor N-hydroxy-7-(2-naphthylthio) heptanomide (HNHA) showed a similar effect on histone H3 and non-histone protein α-tubulin to LBH589 (Kim et al., 2009). HDAC inhibitor TSA significantly reduces sVEGFR1 basal expression, suggesting that histone acetylation is involved in activating sVEGFR1 (Choi et al., 2018). The depletion of HDAC7 in HUVEC results in the overexpression of A-kinase anchor protein 12 (AKAP12), which is a tumor/angiogenesis suppressor gene responsible for the inhibition of migration and tube formation. Mechanistically, H3 histone proteins associated with AKAP12 promoter are highly acetylated following the removal of HDAC7, leading to an increase in its mRNA and protein levels (Turtoi et al., 2012). Intriguingly, regulation of gene locus-specific histone acetylation status by the interaction between a lipid signaling mediator LPA and HDAC7 may downregulate the transcription of angiogenic regulator CD36 via protein kinase D pathway, thereby promoting angiogenesis (Ren et al., 2011, 2016).

### Barrier Function

The endothelial barrier in all organ beds allows the free exchange of water but is restrictive to varying degrees to the transport of solutes (Malik et al., 1989). Functional changes in EC, to a large extent, influence the barrier function. As listed in Table 5, many studies have demonstrated the histone acetylation changes affect the barrier function.

**TABLE 5:** EC barrier function mediated by histone acetylation.

Stimuli	Histone acetylation	HAT or HDAC involved	Targeting	References
Hyperglycemia	H3K9ac		MMP-9 (p65 binding)	Zhong and Kowluru, 2013
	H3K9ac		Claudin-5, Claudin-11	Li et al., 2017
	H3K9ac, H3K14ac	p300, SIRT1	ET-1, TGF-β1	Mortuza et al., 2015
LPS	H3K9ac and H3K18ac		VE-cadherin	Thangavel et al., 2014
Scald injury	H3K9ac		VEGF, MPO	Tang et al., 2018
HDAC inhibitors	H3K9ac, H3K14ac		MDR1 (AHR binding)	You et al., 2019

High glucose impairs the endothelial barrier through multiple histone acetylation mediated signals. Zhong and Kowluru reported that diabetes activates retinal Matrix metalloproteinases (MMP)-9 via LSD1 mediated decrease of histone H3K9 di-methylation. The decreased H3K9 methylation frees up that lysine 9 for acetylation and facilitates the recruitment of p65 to MMP-9 promoter, thus resulting in MMP-9 activation in retinal ECs (Zhong and Kowluru, 2013). Li et al. (2017) demonstrated high glucose suppresses claudins-5 and -11 gene expression in human cardiac microvascular ECs, possibly due to inhibition of acetylated H3K9 in claudin-5 and claudin-11 gene promoters. Mortuza et al. (2015) revealed that high glucose induces p300 expression, and reduces SIRT1 levels, thus increases the acetylation of H3K9 and H3K14, which is attributed to the upregulation of ET1 and TGFβ1. These changes contribute to high glucose-induced endothelial hyperpermeability (Mortuza et al., 2015). In pulmonary ECs, combinatorial treatment with DNA methyltransferase (DNMT) inhibitor Aza and HDAC inhibitor TSA mitigate the LPS increased endothelial permeability due to the enhanced acetylation of H3K9 and H3K18 on the VE-cadherin promoter (Thangavel et al., 2014). HDAC inhibitor VPA treatment significantly alleviates the microvascular permeability and water content of the lung, lowers VEGF and MPO levels, and promotes the acetylation of H3K9 following scald injury (Tang et al., 2018). In brain ECs, multidrug resistance protein 1 (MDR1, ABCB1, P-glycoprotein) is a critical efflux transporter that extrudes chemicals from the blood–brain barrier and limits neuronal exposure to xenobiotics. HDAC inhibitor SAHA upregulates the expression and activity of the MDR1 transporter in immortalized human brain capillary EC hCMEC/D3 by increasing H3K9/K14 acetylation and facilitating aryl hydrocarbon receptor (AHR) binding at the MDR1 promoter (You et al., 2019).

### Thrombosis and Coagulation

Several studies revealed the involvement of histone acetylation modification in EC mediated coagulation and thrombosis (Table 6). Tissue-type plasminogen activator (t-PA) plays a key role in the onset of the fibrinolytic process by converting the zymogen plasminogen into the active enzyme plasmin. Stimulated release of t-PA is pivotal for an intravascular fibrinolytic response and protects the circulation from occluding thrombosis. Arts et al. (1995) reported for the first time that t-PA expression could be regulated by histone acetylation. HDAC inhibitors butyrate, TSA, MS275, and VPA were demonstrated to induce a dose-dependent increase of t-PA expression in ECs, which involves histone H3 and H4 acetylation (Arts et al., 1995; Dunoyer-Geindre and Kruithof, 2011; Larsson et al., 2012). VPA induced t-PA expression is associated with increased acetylation of H3K9, H3K18, H3K23, H3K27, H4K8, and H4K16 at the t-PA promoter (Larsson et al., 2012) and dependent on the proximal GC boxes in the t-PA promoter and may involve interactions with transcription factors Sp2, Sp4, and KLF5 (Ulfhammer et al., 2016). The effect of VPA on t-PA expression was then confirmed *in vivo* (Svennerholm et al., 2014; Larsson et al., 2016). Plasminogen activator inhibitor-1 (PAI-1), the most important serine protease inhibitor of t-PA (Van De Craen et al., 2012), has shown beneficial effects on age-related vascular diseases. PAI-1 is induced in senescent HUVECs and aortas of eld mice. SIRT1 is able to reverse the change by binding to the PAI-1 promoter, resulting in a decreased acetylation of H4K16 at the PAI-1 promoter region (Wan et al., 2014). Clinical study has demonstrated that HDAC inhibitor VPA reduces in exhaustion during repeated and prolonged cumulative stimulated t-PA release and decreases basal PAI-1 levels (Svennerholm et al., 2015), which suggested potentially positive effects of HDAC inhibitors on the expression of t-PA and PAI-1. Von Willebrand factor (vWF), playing an essential role in regulating the balance between blood clotting and bleeding, also is regulated by histone acetylation. Peng et al. (2007) reported that irradiation induces thrombus formation via vWF elevation. The irradiation-caused changes in the association of NF-Y with HDAC1 and PCAF lead to the increased acetylated histone H4 and PCAF recruitment to the vWF promoter, which results in subsequently increased vWF transcription in HUVECs (Peng et al., 2007). Mojiri et al. (2019) reported that, in response to hypoxia, Nuclear Factor IB (NFIB) repressor disassociates from the vWF promoter due to the increased acetylation of the promoter-associated histone H4, and thus increases vWF expression. Tissue factor (TF), another protein involved in coagulation, is demonstrated to lose inducibility during senescence, which occurs following chromatin remodeling at the TF promoter resulting from hypoacetylation of histone H3K9 (Kurz et al., 2014). However, HDAC inhibitors TSA and SAHA were reported to greatly attenuate TF expression induced by TNF-α despite of its effect of promoting histone acetylation (Wang et al., 2007; Hebbel et al., 2010).

**TABLE 6:** Histone acetylation associated with thrombosis and coagulation.

Stimuli	Histone acetylation	HAT or HDAC involved	Targeting	References
HDAC inhibitors	H4ac		t-PA	Arts et al., 1995; Dunoyer-Geindre and Kruithof, 2011
HDAC inhibitors	H3K9ac, H3K18ac, H3K23ac, H3K27ac, H4K8ac, H4K16ac	HDAC3, HDAC5, HDAC7	t-PA	Larsson et al., 2012
Atherosclerosis	H4K16ac	SIRT1	PAI-1	Wan et al., 2014
Irradiation	H4ac	HDAC1, PCAF	vWF	Peng et al., 2007
Hypoxia	H4ac		vWF	Mojiri et al., 2019
Senescence	H3K9ac		TF	Kurz et al., 2014

### Others

Except for vascular tone, inflammation, oxidative stress, angiogenesis, barrier function, and thrombosis and coagulation, histone acetylation is also involved in other EC-associated pathological processes, including endothelial mesenchymal transition (EndMT), senescence, and metabolism. Fu et al. (2009) reported that Notch and TGFβ synergistically upregulate a subset of genes by recruiting Smad3 to both Smad and DNA-binding protein CSL binding sites and cooperatively inducing histone H4 acetylation. Zhang et al. (2016) demonstrated that aging-related transcriptional regulator p49/STRAP alters histone acetylation status and impacts mitochondrial dynamics, thereby reducing mitochondrial function and cardiac performance during mammalian senescence. Zhong et al. found the so-called metabolic memory phenomenon in diabetic retinopathy. This phenomenon is mediated by HDAC activation due to the increased expression of HDAC1, 2, 8 genes and compromised HAT activity, as evidenced by the decreased histone H3 acetylation in the retina and its capillary cells (Zhong and Kowluru, 2010). Pirola et al. (2011) uncovered hyperglycemia-mediated induction of genes and pathways associated with endothelial dysfunction occur through modulation of acetylated H3K9/K14, which is inversely correlated with methyl-CpG content (Pirola et al., 2011). Chen et al. (2010) reported that p300 critically regulates glucose-induced gene expression in ECs via binding to gene promoters and augmenting histone acetylation. In HUVECs, high glucose treatment increases p300 production accompanied by increased binding of p300 to ET-1 and fibronectin promoters and increased transcription of vasoactive factors and extracellular matrix (ECM) proteins (Chen et al., 2010). Tanegashima et al. (2017) demonstrated that fasting-induced production of the ketone body β-hydroxybutyrate (β-OHB) enhances expression of the glucose transporter gene Slc2a1 (Glut1) via HDAC2 mediated H3K9 acetylation at the critical cis-regulatory region of the Slc2a1 gene in brain microvascular ECs.

## Conclusion and Future Perspective

**FIGURE 4:**
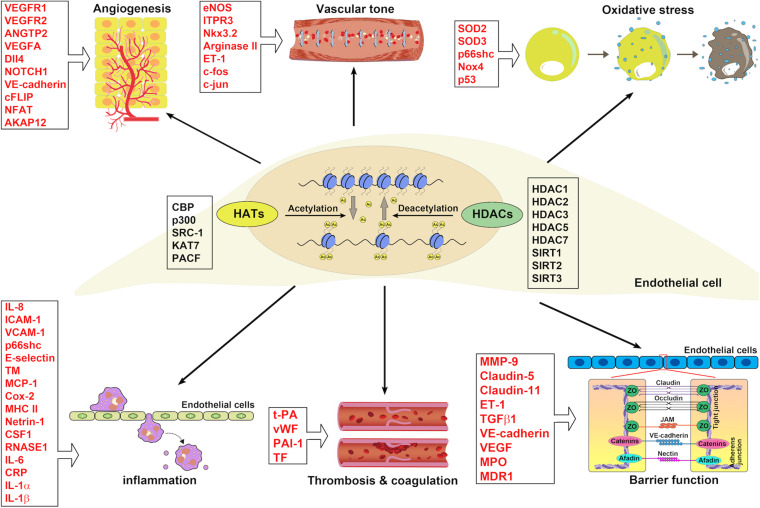
The implications of HATs and HDACs in ECs associated with blood vessel functions. HATs and HDACs have been demonstrated to modulate the histone acetylation of ECs involving blood vessel functions: angiogenesis, vascular tone, oxidative stress, inflammation, thrombosis and coagulation, and barrier function.

Histone acetylation has been found to be involved in the pathogenesis of EC dysfunction in the last two decades. The acetylation of histone at different lysine residues alters under various stimuli and affects gene expression in ECs (Figure 4), therefore mediating endothelial functions. In regards to acetylated histone-mediated gene expression, HATs and HDACs are indispensable. The fact that histone acetylation can be reversed makes it an ideal target for drug treatment. With the development of HDAC inhibitors, which could inhibit HDACs, therefore, promoting histone acetylation, modulation of histone acetylation becomes achievable. In the oncology field, HDAC inhibitors have already been tested in clinical trials for treating solid and hematological malignancies (O’Connor et al., 2015; Sekeres et al., 2017; Tambo et al., 2017; Gray et al., 2019; Rodriguez et al., 2020).

The application of HDAC inhibitors in EC dysfunction-related disease is promising but still in its infantile stage. Many challenges are awaiting to be conquered. The side effects of HDAC inhibitors on the acetylation of non-histone proteins and other non-acetylation effects may limit their application. For example, the non-selective HDAC inhibitor vorinostat, inhibiting the activities of class I and class II HDAC enzymes, potentially increases the lysine acetylation status of more than 1,700 non-histone proteins (Choudhary et al., 2009). HDAC inhibitor LBH589 and HNHA have an acetylation effect on non-histone protein α-tubulin (Qian et al., 2006; Kim et al., 2009). HDAC inhibitor TSA could acetylate non-histone protein p53 (Zeng et al., 2003) and has ubiquitination effect on histone acetyltransferase p300 (Hakami et al., 2016). HDAC inhibitor VPA could induce ERK1/2 phosphorylation independent of the HDAC inhibition effect (Michaelis et al., 2006). In ECs, except for the histone deacetylation effect, HDACs were also reported to deacetylate non-histone proteins. HDAC1 was demonstrated to deacetylate p53 and transactivate the p21 under laminar flow (Zeng et al., 2003). SIRT1 activates the expression of antioxidant genes in ECs by deacetylating and increasing the activity of the transcription factors FOXO3a and PGC-1α (Olmos et al., 2013). Furthermore, many HDACs form corepressor complexes to silence gene activity, which again amplifies the potential non-specific effects that HDAC inhibitors can have within the cell (Krishna et al., 2015). Therefore, there is a critical need for further investigation to elucidate the roles of HDACs and HATs in regulating the acetylation of non-histone proteins in ECs.

Not all the HATs and HDACs, which are potentially involved in endothelial dysfunction-related histone acetylation, have been elucidated. The systemic study using individual HATs and HDACs endothelial-specific knockout mice should be essential for filling up our current knowledge gaps. Same HAT or HDAC may regulate different genes that have beneficial or detrimental EC functions in various physiological and pathological settings. HDAC1 has an anti-inflammatory effect via histone acetylation mediated IL-8 expression (Jiang et al., 2015) and pro-oxidative stress through SOD3 as well (Ohashi et al., 2017). Though HDAC2 could decrease pro-inflammatory gene expression (Liu et al., 2016), it also inhibits anti-oxidative stress gene SOD3 expression (Hou et al., 2018). It could be another challenge in how to target HDAC mediated histone acetylation for modulating endothelial functions precisely. In addition, histone acetylation and methylation always work together. The complexity of histone posttranslational modification in ECs needs our endeavor to be revealed in the future.

## Author Contributions

QM provided the outline of the manuscript. ZF, XW, and XS searched the literature for the article and wrote the manuscript. WH coordinated and revised the content and prepared the figures. ZF prepared the tables. QM supervised the work and completed the final editing. All authors discussed its content and edited the submitted manuscript.

## Conflict of Interest

The authors declare that the research was conducted in the absence of any commercial or financial relationships that could be construed as a potential conflict of interest.
